# Effects of a recess-based ocular muscle regulation training program on visual function in adolescents: a school-based controlled study

**DOI:** 10.3389/fpubh.2026.1812557

**Published:** 2026-04-29

**Authors:** Hongxiu Chen, Xingze Wang, Guoqiang Cai, Yicheng Yang, Yuan Xie

**Affiliations:** 1 Department of Exercise and Sport Sciences, Faculty of Graduate School, Khon Kaen University, Khon Kaen, Thailand; 2 School of Physical Education, Huzhou University, Zhejiang, China; 3 Huzhou No. 2 High School, Huzhou, Zhejiang, China; 4 Sichuan University of Media and Communications, Sichuan, China; 5 Heilongjiang University of Technology, Heilongjiang, China

**Keywords:** adolescent myopia, noncycloplegic refraction, ocular muscle regulation training, school-based intervention, visual function

## Abstract

**Objective:**

This study aimed to investigate the effects of a 16-week recess-based ocular muscle regulation training program (OMRTP) on uncorrected visual acuity (VA) and noncycloplegic spherical equivalent (SE) in adolescents.

**Methods:**

This school-based controlled study enrolled Grade 10–11 students (U15–U16; *n* = 200) from Huzhou No. 2 High School in Huzhou, China. Students were assigned at the class-cluster level within the same school to an experimental group (EG, *n* = 100) or a control group (CG, *n* = 100). The experimental group completed a 16-week recess-based OMRTP, whereas the control group received routine vision health education only. VA and noncycloplegic SE were assessed at baseline (week 0) and post-intervention (week 17). Intervention effects were evaluated using linear mixed-effects models within a difference-in-differences (DiD) framework.

**Results:**

Baseline VA and SE were comparable between groups (all *P* > 0.05). For SE, the EG demonstrated more favorable short-term functional shifts in noncycloplegic SE relative to the CG across age strata and eyes (DiD: U15 left 0.318 D, right 0.358 D; U16 left 0.268 D, right 0.210 D; all *P* < 0.001). For VA, the EG showed greater improvements than the CG across age strata and eyes (DiD: U15 left 0.115, right 0.074; U16 left 0.156, right 0.105; all *P* < 0.05). Overall, mixed-effects models confirmed a significant group × time interaction for both SE (*F* = 62.08, *P* < 0.001) and VA (*F* = 42.03, *P* < 0.001).

**Conclusions:**

A 16-week recess-based OMRTP was associated with improvements in uncorrected visual acuity and short-term functional changes in noncycloplegic spherical equivalent refraction among adolescents.

## 1 Introduction

The prevalence of myopia is increasing at an unprecedented rate worldwide, with the highest burden observed in East Asia (1, 2). Projections suggest that by 2050, approximately 50% of the global population will be affected by myopia, with around 10% progressing to high myopia (3). This trend is expected to substantially increase the risk of vision-threatening complications, including myopic maculopathy, retinal detachment, and glaucoma, as well as irreversible visual impairment (3). In China, the prevalence of myopia among children and adolescents remains consistently high, often exceeding 80% at the senior high school level. Such a substantial disease burden not only adversely affects academic performance and quality of life but also imposes considerable long-term socioeconomic costs (4).

The onset and progression of myopia are closely associated with axial elongation and alterations in the refractive system (5). Although genetic factors contribute to individual susceptibility, their explanatory power remains limited, suggesting that the recent rapid increase in myopia prevalence is more strongly linked to changes in environmental exposures and lifestyle behaviors (6, 7). Childhood and adolescence represent critical periods of ocular development, during which increasing academic demands and prolonged near-work activities place individuals at heightened risk of myopia onset and progression (8). From a mechanistic perspective, sustained high-intensity near work requires continuous accommodative effort and is associated with accommodative response errors (e.g., accommodative lag) as well as alterations in accommodative dynamics. These changes may increase retinal defocus and contribute to visual fatigue (9). Accommodative lag may also be accompanied by transient axial elongation and choroidal changes, potentially generating pro-myopic biological signals in susceptible individuals; however, the causal role of these mechanisms in human myopia development remains under debate (10). In parallel, exposure to high-illuminance natural light in outdoor environments has been identified as a protective factor, likely mediated through light-dependent retinal signaling pathways, particularly dopaminergic mechanisms, which are involved in the negative regulation of axial eye growth. Insufficient outdoor exposure may attenuate this protective effect (11–14). In addition, a subset of adolescents exhibits binocular vision anomalies, such as accommodative insufficiency or reduced fusional capacity, which may further increase visual load and be associated with myopia progression (15).

In school settings, the U15–U16 stage is characterized by increased curriculum intensity and prolonged near-work exposure, which may place greater demands on the accommodative system and is associated with visual fatigue. Limited outdoor exposure during recess further reduces opportunities for distance viewing and natural light stimulation, potentially contributing to sustained near-visual load and the development of maladaptive visual behaviors (14, 16). In this context, current myopia control strategies are commonly classified into optical, pharmacological, and behavioral or environmental interventions (5). Optical and pharmacological approaches typically require individualized prescriptions, ongoing follow-up, and medical supervision; consequently, schools primarily assume responsibilities related to health education, vision screening, and referral pathways for further clinical management (17, 18). By contrast, behavioral and environmental interventions, together with standardized accommodative or visual function training that can be implemented in group settings, may be more feasible within school environments and align with institutional roles (5, 19).

The integrated ocular muscle regulation training program (OMRTP) represents a structured intervention combining accommodative training, oculomotor function training, and implementation in outdoor or well-illuminated environments. First, accommodative training involving alternating near–distance fixation with distance relaxation facilitates dynamic accommodative adjustments and enhances the stability and flexibility of accommodative responses (16, 20, 21). Second, training targeting saccades, pursuit, and fixation stability improves oculomotor control efficiency and binocular coordination, thereby reducing visual load during academic tasks (22). Third, delivery in outdoor or well-illuminated settings increases exposure to natural light and interrupts prolonged near-work activities (14). Collectively, these components are expected to act synergistically to improve visual function and reduce near-work–related visual burden, providing a feasible framework for school-based interventions.

The present study aimed to evaluate the effects of this intervention on uncorrected visual acuity (VA) and noncycloplegic spherical equivalent (SE) in U15–U16 adolescents. We hypothesized that, compared with routine vision health education, the OMRTP would significantly improve VA and induce favorable changes in SE.

## 2 Materials and methods

This study was approved by the Ethics Review Committee of Huzhou University (Approval No. 202502-01, February 17, 2025). All procedures were conducted in accordance with the Declaration of Helsinki. The study was carried out at Huzhou No. 2 High School in Huzhou, China. Written informed consent was obtained from the parents or legal guardians of all participants prior to participation, and assent was obtained from the students themselves. Permission to conduct the study was also granted by the participating school.

### 2.1 Participants

Participants were Grade 10–11 students (approximately 15–16 years) recruited from Huzhou No. 2 High School in Huzhou, China. This grade range was selected because students typically experience high near-work demands and elevated visual load, while limiting heterogeneity in ocular development that may occur across broader age spans. Recruitment was conducted at the class-cluster level within the same school: one Grade 10 class and one Grade 11 class were allocated to the experimental group, and one parallel Grade 10 class and one Grade 11 class were allocated to the control group (approximately 50 students per class). Of 206 students screened, 203 met the eligibility criteria and were enrolled (EG, *n* = 101; CG, *n* = 102). The sample size was determined based on the actual number of eligible students within the school and the feasibility of implementing a class-cluster-based intervention. Participants were allocated at the class level to facilitate standardized intervention delivery and management in the school setting. A formal *a priori* statistical power analysis was not conducted.

Eligible participants were able to complete visual acuity and refractive assessments and to participate in the intervention as required. To preserve feasibility and real-world representativeness in this school-based program, no strict refractive error thresholds were applied; students with a range of baseline refractive profiles were included. Baseline SE was included as a covariate in the statistical models. Exclusion criteria included ocular organic disease or conditions affecting visual function; suspected or confirmed pathological-myopia-related complications or other fundus abnormalities requiring medical referral; high hyperopia (≥ +6.0 D); high astigmatism (cylinder magnitude ≥ 3.0 D); and inability to participate as required. Three students were excluded (one ineligible; two declined or had incomplete guardian consent).

**Figure 1:**
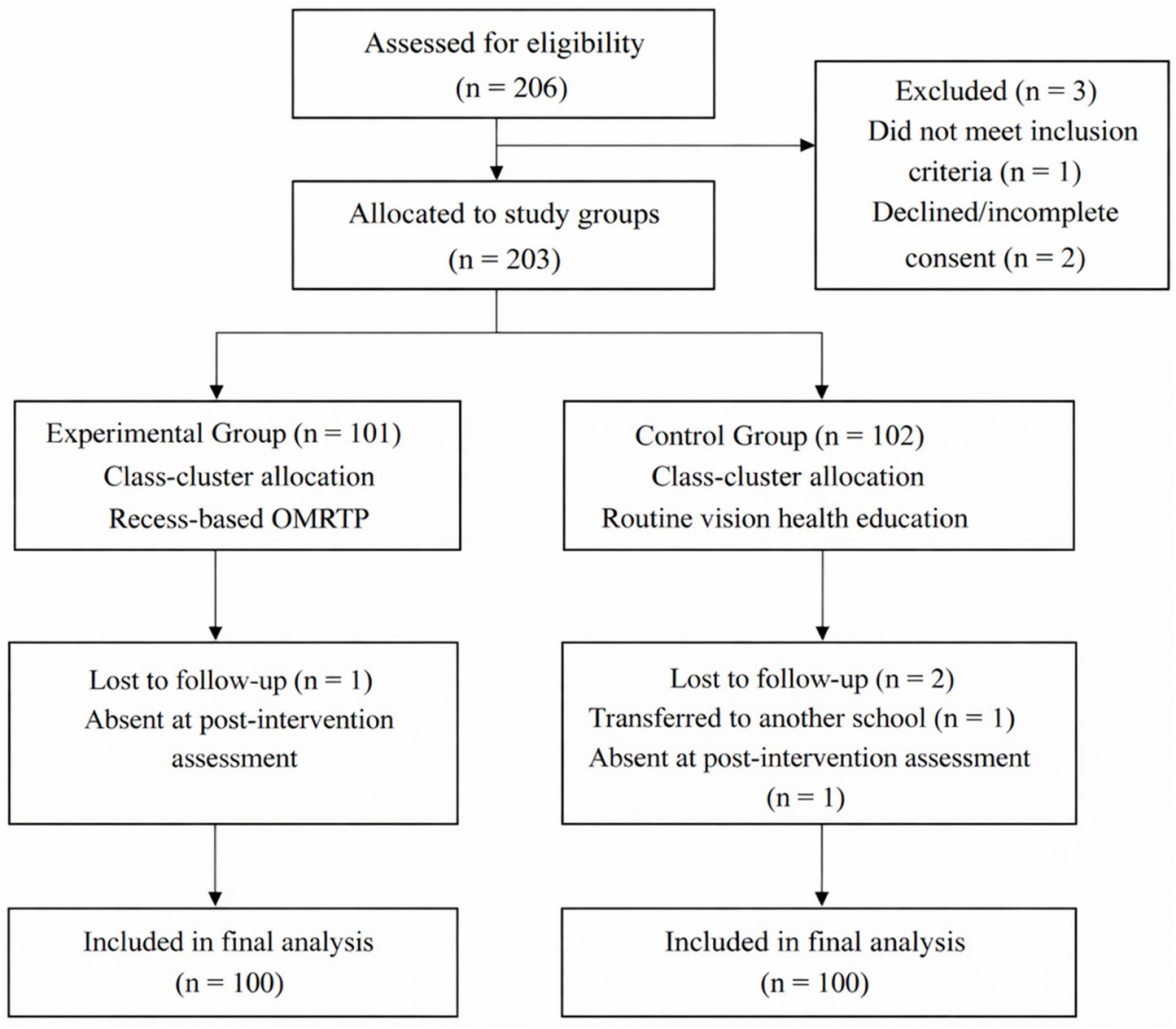
Participant flow diagram. The figure was created by the authors using Microsoft Word (Microsoft 365, Version 16.100.4, Microsoft Corporation, USA).

Group assignment followed a within-school class-cluster structure. Because allocation was conducted at the class level rather than through individual randomization, residual class-level differences (e.g., classroom environment or teacher-related factors) cannot be completely ruled out. Therefore, findings should be interpreted as evidence from a controlled school-based study and warrant confirmation in larger multi-class or multi-school cluster-randomized trials. Because the intervention was delivered in a school setting, blinding of students and implementers was not feasible; however, outcome examiners were blinded to group assignment, and statistical analyses were conducted using anonymised group codes. After the intervention, one participant in the experimental group and two in the control group were lost to follow-up, resulting in 100 participants per group for analysis (Figure 1). Adverse events were monitored throughout; participants could pause or withdraw if discomfort occurred (e.g., dizziness, eye pain, tearing). The study followed the Declaration of Helsinki, and written informed consent was obtained from students and their parents or legal guardians prior to enrolment.

### 2.2 Intervention

**Figure 2:**
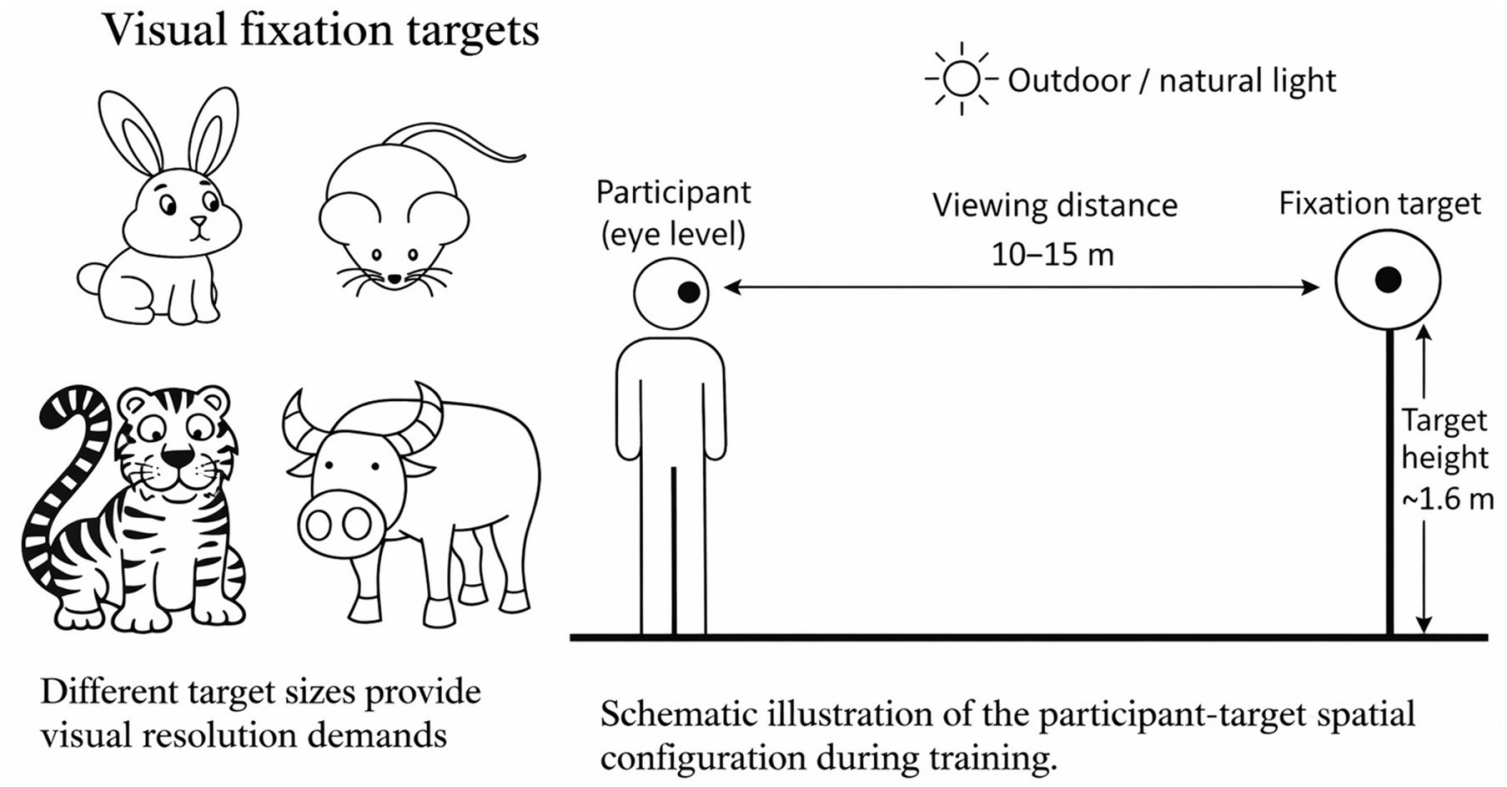
Recess-based visual fixation target training conducted in an outdoor school setting. Left: examples of visual fixation targets with graded sizes. Right: schematic illustration of the participant–target setup, illustrating the prescribed viewing distance (10–15 m) and target height (1.6 m) during recess-based training under natural light conditions. The figure was created by the authors using Microsoft Word (Microsoft 365, Microsoft Corporation, USA).

**Figure 3:**
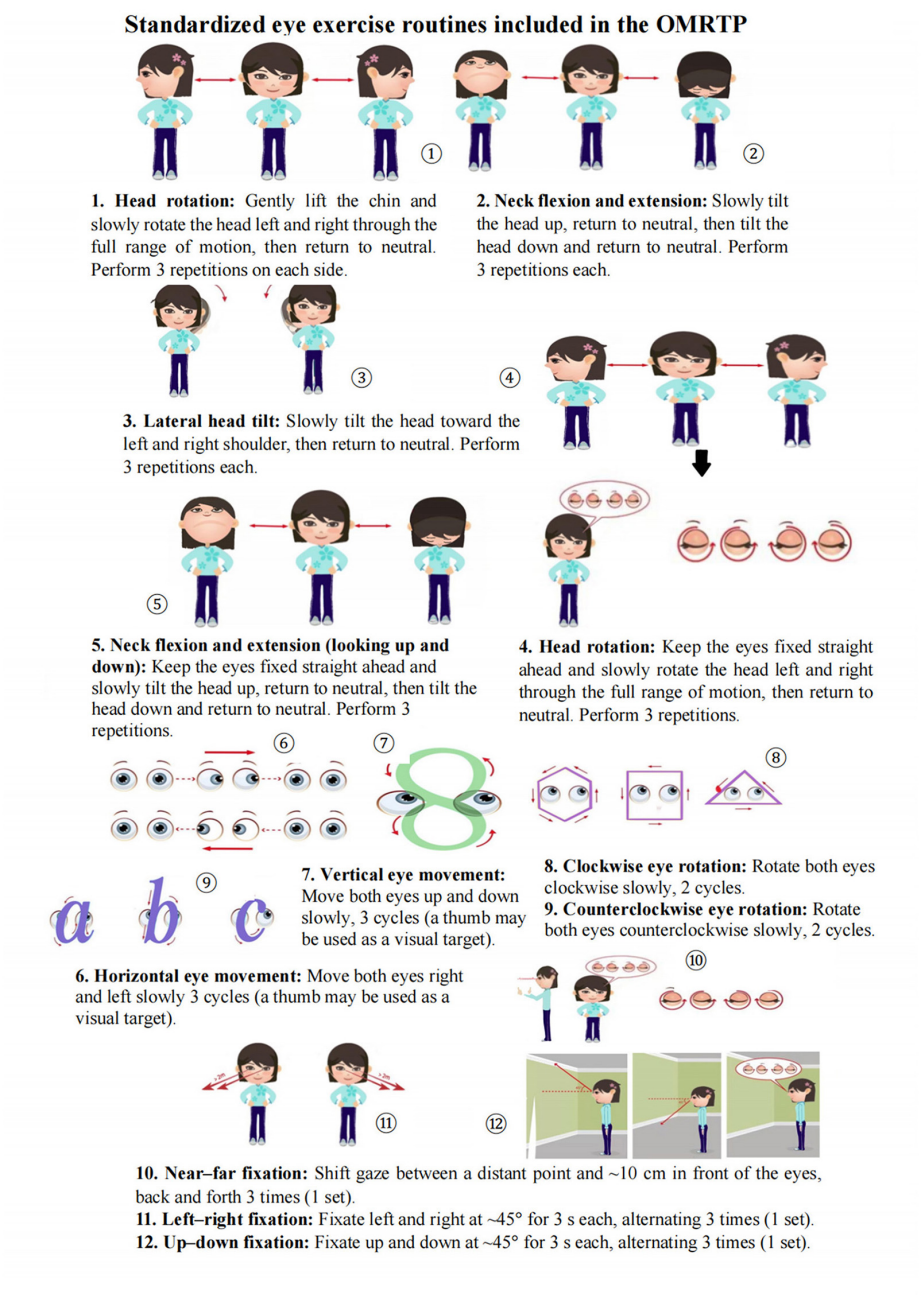
Eye exercise routines performed during the recess-based OMRTP. The routines are performed sequentially from exercises 1–12 under teacher supervision, with standardized verbal instructions to ensure consistent execution across participants. The protocol is organized into three components: (1) head and neck movements (exercises 1–5), (2) eye movement training (exercises 6–9), and (3) gaze fixation exercises (exercises 10–12). Each session lasts approximately 5 min and is performed collectively during school recess. The figure illustrates the correct posture, movement direction, and repetition pattern for each exercise to facilitate reproducibility. The figure was created by the authors using Microsoft Word (Microsoft 365, Microsoft Corporation, USA).

**Figure 4:**
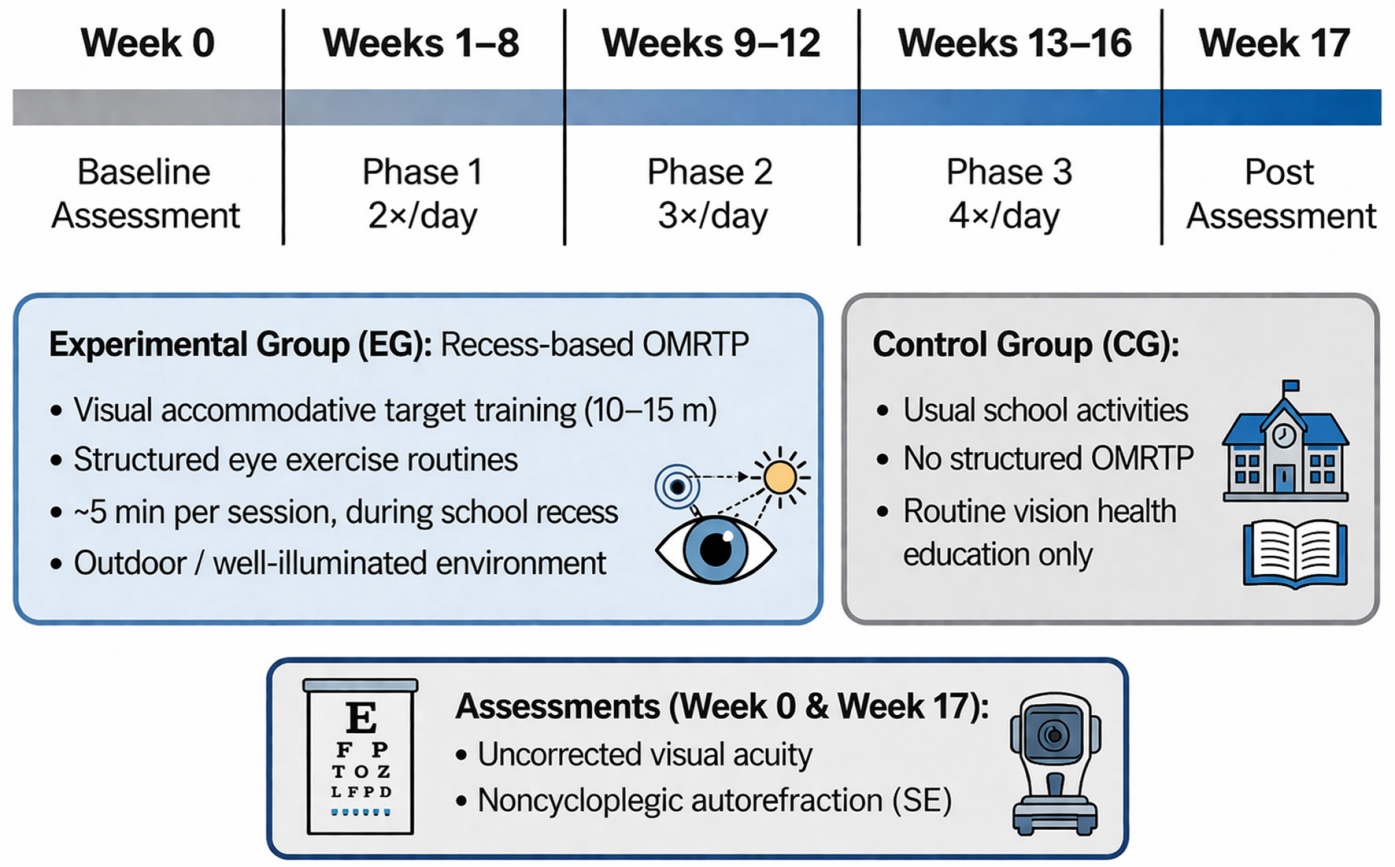
Timeline of the 16-week recess-based OMRTP intervention. The figure was created by the authors using Microsoft Word (Microsoft 365, Microsoft Corporation, USA).

Group allocation followed a within-school class-cluster structure at Huzhou No. 2 High School. Two classes (one Grade 10 and one Grade 11) were allocated to the experimental group (EG), and two parallel classes (one Grade 10 and one Grade 11) were allocated to the control group (CG). Allocation was conducted at the class level without randomization, and outcome assessors were blinded to group codes. The EG completed a 16-week recess-based OMRTP delivered under the unified organization and supervision of trained personnel. The OMRTP comprised two standardized components: visual accommodative target training (Figure 2) and structured eye exercise routines (Figure 3). The program followed a progressive schedule with stepwise increases in daily frequency across three phases (Figure 4): Weeks 1–8, twice daily; Weeks 9–12, three times daily; and Weeks 13–16, four times daily. Each session lasted approximately 5 min. All sessions were scheduled during school recess to facilitate distance viewing and exposure to natural light; students left the classroom and performed the program in open areas such as the school playground or well-illuminated corridors. The CG continued usual school activities and did not receive the structured OMRTP during the study period. To ensure ethical fairness, the CG received routine school-based vision health education (e.g., near-work hygiene, reading and writing posture, regular study breaks, and the importance of outdoor activity).

Outcome assessments were conducted at Week 0 (baseline) and Week 17 (post-intervention), and primary analyses were based on these two time points. Uncorrected visual acuity was measured under standardized lighting using a 5-m visual acuity chart and recorded in decimal notation. Noncycloplegic refractive measurements were obtained using an autorefractor (NIDEK ARK-1, NIDEK Co., Ltd., Japan); each eye was measured three times and averaged. Spherical equivalent refraction was calculated as SE = sphere + 0.5 × cylinder (diopters). All assessments were performed by ophthalmologists following standardized operating procedures, with consistent test environment, viewing distance, instructions, and fixed test sequence; outlying values were remeasured for confirmation.

#### 2.2.1 Visual accommodative target training

Visual accommodative target training was conducted during school recess. To provide broader viewing distances, reduce continuous near-work load in the classroom, and increase exposure to natural light, students performed the training in designated open areas outside the classroom. The protocol involved alternating near and distance fixation, with distance relaxation incorporated into each cycle to facilitate accommodative flexibility. Each session lasted approximately 5 min and consisted of three consecutive cycles. Each cycle included 1 min of target fixation followed by approximately 30 s of distance relaxation, ensuring feasibility within short recess periods and enabling repeated near–distance fixation transitions. Visual targets were positioned at approximately 10–15 m and a height of 1.6 m; this viewing distance was selected to approximate optical infinity and promote accommodative relaxation. Targets of varying sizes (21 × 21 cm, 18 × 18 cm, 15 × 15 cm, and 10 × 10 cm) were used to provide graded levels of visual discrimination demand, thereby progressively increasing visual acuity requirements and promoting active accommodative engagement during training. In addition, the visual targets were designed using simple, high-contrast, and familiar shapes with recognizable features to facilitate stable fixation, enhance visual attention, and support effective visual discrimination during training. The training incorporated repeated switching between near and distance visual tasks combined with sustained fixation, which may improve visual coordination and accommodative function (23, 24).

#### 2.2.2 Eye exercise routines

The eye exercise routines used in this study consisted of a structured set of ocular movements. The content and organization of the exercises was adapted from eye exercise programs widely implemented in school settings in China and was standardized for the purposes of the present study (25). The routines were performed during school recess under the unified organization and supervision of trained personnel. In total, the program comprised 12 exercises arranged into three components, which were performed sequentially from Exercise 1 to Exercise 12 to ensure consistent execution across participants. The first component involved head and neck movements, including exercises performed both without and with fixed gaze (horizontal head rotation, vertical nodding, and lateral head tilting, as well as head movements while maintaining fixation on a stationary target). The second component included eye movement training, specifically horizontal and vertical eye movements as well as clockwise and counterclockwise circular rotations. The third component focused on gaze fixation exercises, incorporating near and distance fixation as well as left, right, upward, and downward gaze fixation. Each exercise was performed according to a predefined number of repetitions, with a total duration of approximately 5 min per session. Participants were instructed to discontinue the exercises immediately if symptoms such as dizziness, eye pain, or tearing occurred. Any adverse events were recorded, and participants withdrew from the study if necessary. Compliance was monitored throughout the 16-week intervention period. Attendance and participation were recorded for each session by designated supervising teachers assigned to each class. Weekly adherence was reviewed, and any absences or missed sessions were documented. The intervention was delivered in a standardized, class-based format under teacher supervision, which facilitated consistent implementation and supported adherence throughout the intervention period.

## 3 Statistical analysis

All statistical analyses were performed using R software (R Foundation for Statistical Computing, Vienna, Austria). Linear mixed-effects models were constructed separately for uncorrected visual acuity, expressed as decimal VA, and spherical equivalent refraction (SE). For statistical modeling, uncorrected visual acuity was additionally analyzed on the logarithm of the minimum angle of resolution (logMAR) scale to improve linearity and better meet model assumptions; all statistical inferences were consistent across both scales. Participant ID was specified as a random intercept to account for within-subject correlations arising from repeated measurements over time and from measurements of both eyes. Because allocation was conducted at the class-cluster level within a single school and the number of class clusters per group was limited, inclusion of class-level random effects was not statistically robust; therefore, analyses were performed at the individual level and findings should be interpreted with potential residual class-level confounding in mind. Fixed effects included age group (U15, U16), group (EG, CG), time (pre-intervention, post-intervention), eye (left, right), and sex. All theoretically relevant two-way and three-way interaction terms were included, including the age × group × time interaction. Statistical significance of fixed effects was assessed using Type III F tests with Satterthwaite approximation for degrees of freedom. Effect sizes were reported as partial eta squared (η*p*^2^). Estimated marginal means and corresponding 95% confidence intervals were calculated using the emmeans package in R. Intervention effects were evaluated primarily through the group × time interaction term. In addition, a difference-in-differences framework was applied to compare changes from baseline to post-intervention between the experimental and control groups, with adjusted difference-in-differences estimates reported separately for the U15 and U16 age strata.

## 4 Results

A total of 200 students were included in the final analysis, with 100 participants in each group. The experimental group (EG) included 55 males and 45 females, and the control group (CG) included 52 males and 48 females. Baseline sex distribution, grade composition, and baseline values of uncorrected visual acuity and spherical equivalent refraction in both eyes did not differ significantly between groups (all *P* > 0.05), indicating comparable baseline characteristics.

### 4.1 Spherical equivalent refraction

**Figure 5:**
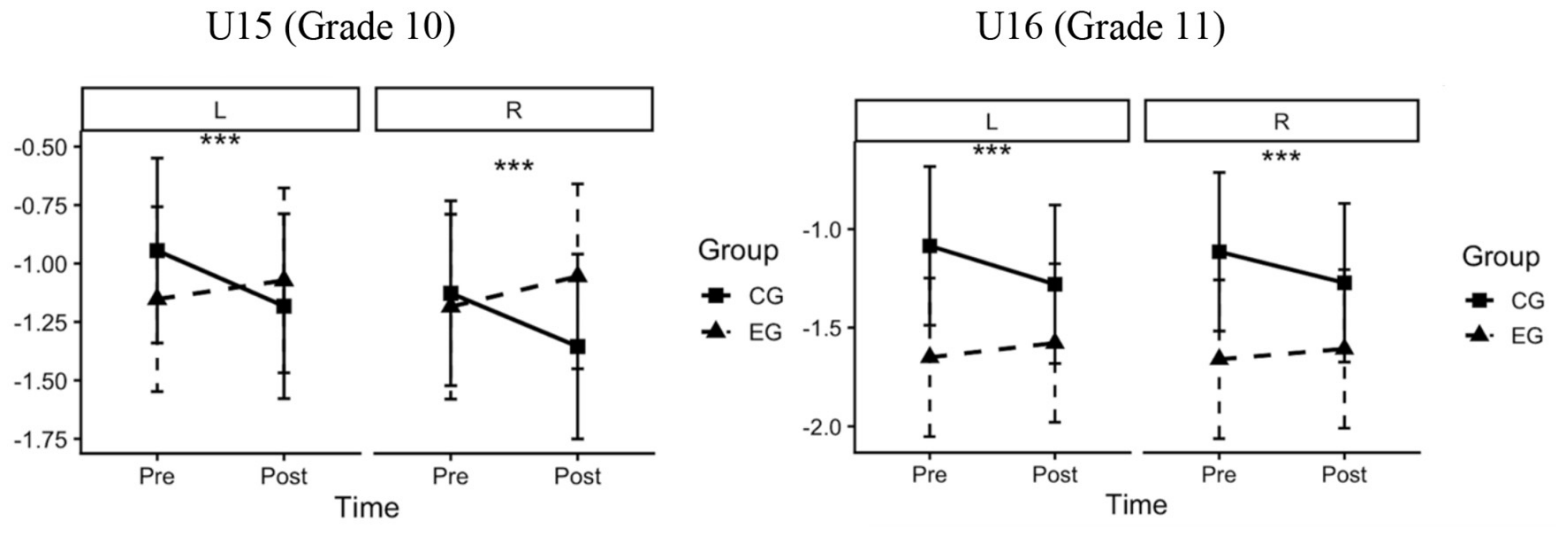
Difference-in-differences estimates for SE between the EG and CG in the pooled sample combining U15 (Grade 10) and U16 (Grade 11) students. Panels show the left **(L)** and right **(R)** eyes. Lines depict model-estimated marginal means from linear mixed-effects models including fixed effects for group, time (pre-intervention vs. post-intervention), and their interaction, adjusted for baseline SE, age, sex, and other covariates. Error bars indicate 95% confidence intervals. Statistical significance of the group × time interaction is indicated as *P* < 0.001 (***). SE, spherical equivalent refraction (D). The figure was produced using R (R Foundation for Statistical Computing, Vienna, Austria).

Descriptive statistics for SE (D) are presented in Table 1. Overall, mean noncycloplegic SE became less negative in the EG across age strata and eyes, whereas it became more negative in the CG over the same period. Difference-in-differences mixed-effects models showed more favorable changes in SE in the EG than in the CG in both age strata and eyes (Table 2). In the U15 stratum, the adjusted between-group difference was 0.318 D for the left eye (95% CI, 0.134–0.501; *F* = 11.56; η*p*^2^ = 0.038; *P* < 0.001) and 0.358 D for the right eye (95% CI, 0.174–0.541; *F* = 14.65; η*p*^2^ = 0.047; *P* < 0.001). In the U16 stratum, the adjusted between-group difference was 0.268 D for the left eye (95% CI, 0.180–0.355; *F* = 36.22; η*p*^2^ = 0.110; *P* < 0.001) and 0.210 D for the right eye (95% CI, 0.123–0.297; *F* = 22.32; η*p*^2^ = 0.071; *P* < 0.001). In the overall mixed-effects model, the group × time interaction was significant (*F* = 62.08; *P* < 0.001; η*p*^2^ = 0.095), indicating differential change in SE between groups from baseline to post-intervention. Significant main effects of time (*F* = 10.88; *P* = 0.001; η*p*^2^ = 0.018) and eye (*F* = 8.74; *P* = 0.003; η*p*^2^ = 0.015) were also observed, whereas the age × group × time interaction was not significant (*P* > 0.05) (Table 3). Consistent with these findings, the model-estimated marginal means showed divergent pre–post trajectories in SE between the EG and CG for both eyes in the pooled Grade 10–11 sample (Figure 5).

**Table 1:** Descriptive statistics of spherical equivalent at pre and post.

Age group	Eye	Group	Pre (mean ±SD)	Post (mean ±SD)
U15	Left	EG	−1.15 ± 1.42	−1.07 ± 1.47
		CG	−0.95 ± 1.40	−1.18 ± 1.43
Right	EG	−1.19 ± 1.43	−1.06 ± 1.47
	CG	−1.13 ± 1.45	−1.36 ± 1.42
U16	Left	EG	−1.65 ± 1.61	−1.58 ± 1.66
		CG	−1.09 ± 1.16	−1.28 ± 1.27
Right	EG	−1.66 ± 1.58	−1.61 ± 1.61
	CG	−1.12 ± 1.14	−1.28 ± 1.23

Values are presented as mean ± SD. SE, spherical equivalent refraction (D). U15 and U16 denote Grade 10 and Grade 11, respectively. Pre and post indicate measurements before and after the intervention. EG, experimental group; CG, control group.

**Table 2:** Difference-in-differences (EG – CG) in SE estimated by linear mixed-effects models.

Age group	Outcomes	Adj. mean diff. 95% CI	*F*	*ηp* ^2^	*P*
U15	SE-L	0.318 (0.134–0.501)	11.56	0.038	<0.001
	SE-R	0.358 (0.174–0.541)	14.65	0.047	<0.001
U16	SE-L	0.268 (0.180–0.355)	36.22	0.110	<0.001
	SE-R	0.210 (0.123–0.297)	22.32	0.071	<0.001

DiD estimates (EG – CG) were obtained from linear mixed-effects models; the adjusted mean difference corresponds to the group × time interaction, with 95% confidence intervals reported for each estimate. Models included a random intercept for participant ID and were adjusted for baseline SE and age. SE-L, left eye; SE-R, right eye; SE, spherical equivalent refraction (D).

**Table 3:** Linear mixed-effects model for SE (Age × Group × Time).

Effect	*F*	*P*	η*p*^2^
Age	1.858	0.174	0.009
Group	0.875	0.351	0.004
Time	10.879	0.001	0.018
Eye	8.743	0.003	0.015
Sex	1.791	0.182	0.009
Group × Time	62.077	<0.001	0.095
Age × Group × Time	1.823	0.177	0.003

Linear mixed-effects models were fitted with Age (U15, U16), Group (EG, CG), Time (pre-intervention, post-intervention), Eye (left, right), and Sex as fixed effects, with participant ID as a random intercept. Fixed effects were evaluated using Type III F tests with Satterthwaite approximation. Effect sizes are reported as partial eta squared (η*p*^2^).

### 4.2 Visual acuity

**Figure 6:**
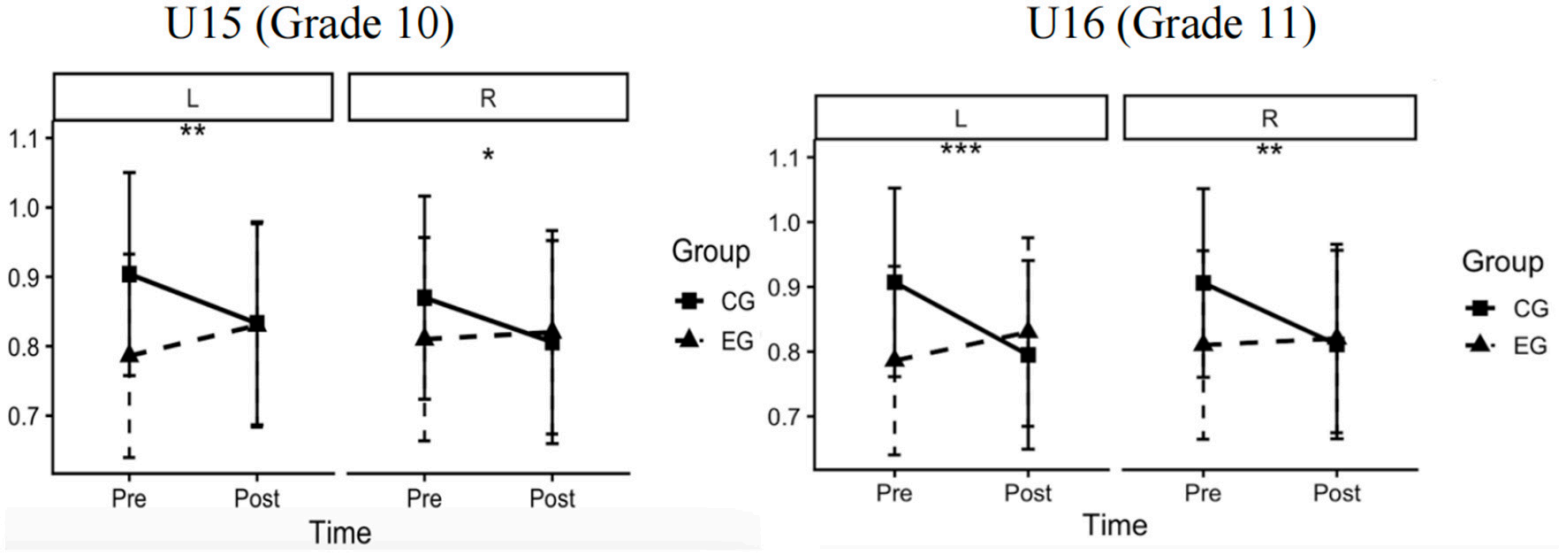
Difference-in-differences estimates for uncorrected visual acuity between the EG and CG in the pooled sample combining U15 (Grade 10) and U16 (Grade 11) students. Panels show the left **(L)** and right **(R)** eyes. Lines depict estimated marginal means derived from linear mixed-effects models with fixed effects for group, time (pre-intervention vs. post-intervention), and their interaction, adjusted for baseline visual acuity, age, and other covariates. Error bars indicate 95% confidence intervals. Statistical significance of the group × time interaction is indicated as *P* < 0.05 (*), *P* < 0.01 (**), and *P* < 0.001 (***). The figure was produced using R (R Foundation for Statistical Computing, Vienna, Austria).

Descriptive statistics for uncorrected decimal VA are presented in Table 4. Mean VA increased after the intervention in both eyes in the EG across both age strata, whereas no consistent improvement was observed in the CG over the same period. Difference-in-differences mixed-effects models showed significantly greater improvements in VA in the EG than in the CG for both age strata and eyes (Table 5). In the U15 stratum, the adjusted between-group difference was 0.115 for the left eye (95% CI, 0.05–0.18; *F* = 10.76; η*p*^2^ = 0.035; *P* = 0.001) and 0.074 for the right eye (95% CI, 0.01–0.14; *F* = 4.46; η*p*^2^ = 0.015; *P* = 0.036). In the U16 stratum, the adjusted between-group difference was 0.156 for the left eye (95% CI, 0.09–0.22; *F* = 20.62; η*p*^2^= 0.066; *P* < 0.001) and 0.105 for the right eye (95% CI, 0.04–0.17; *F* = 9.34; η*p*^2^ = 0.031; *P* = 0.002). In the overall mixed-effects model, the group × time interaction was significant (*F* = 42.03; *P* < 0.001; η*p*^2^ = 0.067), accompanied by a significant main effect of time (*F* = 11.37; *P* < 0.001; η*p*^2^ = 0.019). No other main effects or interaction terms, including age, sex, and eye, reached statistical significance (all *P* > 0.05) (Table 6). As a robustness check, the same models were also evaluated on the logMAR scale, and the direction and statistical significance of the group × time effects remained unchanged (Table 7). Consistent with these findings, the model-estimated marginal means demonstrated divergent pre-post trajectories of uncorrected VA between the EG and CG for both eyes in the pooled Grade 10–11 sample (Figure 6).

**Table 4:** Descriptive statistics of decimal visual acuity at pre and post.

Age group	Eye	Group	Pre (mean ±SD)	Post (mean ±SD)
U15	Left	EG	0.78 ± 0.31	0.83 ± 0.32
		CG	0.90 ± 0.33	0.83 ± 0.34
Right	EG	0.80 ± 0.30	0.84 ± 0.31
	CG	0.88 ± 0.32	0.81 ± 0.33
U16	Left	EG	0.78 ± 0.50	0.83 ± 0.54
		CG	0.91 ± 0.51	0.80 ± 0.49
Right	EG	0.81 ± 0.54	0.82 ± 0.53
	CG	0.91 ± 0.51	0.81 ± 0.51

Values are presented as mean ± SD. VA, uncorrected decimal visual acuity; higher values indicate better visual acuity. U15 and U16 denote Grade 10 and Grade 11, respectively. EG, experimental group; CG, control group.

**Table 5:** Difference-in-differences (EG – CG) in decimal visual acuity estimated by linear mixed-effects models.

Age group	Outcomes	Adj. mean diff. 95% CI	*F*	*η*p** ^2^	*P*
U15	VA-L	0.115 (0.05–0.18)	10.76	0.035	0.001
	VA-R	0.074 (0.01–0.14)	4.46	0.015	0.036
U16	VA-L	0.156 (0.09–0.22)	20.62	0.066	<0.001
	VA-R	0.105 (0.04–0.17)	9.34	0.031	0.002

DiD estimates (EG – CG) were obtained from linear mixed-effects models; the adjusted mean difference corresponds to the group × time interaction, with 95% confidence intervals reported for each estimate. Models included a random intercept for participant ID and were adjusted for baseline visual acuity and age. VA-L, left eye; VA-R, right eye.

**Table 6:** Linear mixed-effects model for visual acuity (age × group × time).

Effect	*F*	*P*	η*p*^2^
Age	0.000	0.992	<0.001
Group	0.346	0.557	0.002
Time	11.366	<0.001	0.019
Eye	0.067	0.795	<0.001
Sex	2.048	0.154	0.01
Group × Time	42.032	<0.001	0.067

Linear mixed-effects models were fitted with Age (U15, U16), Group (EG, CG), Time (pre-intervention, post-intervention), Eye (left, right), and Sex as fixed effects, with participant ID as a random intercept. Fixed effects were evaluated using Type III F tests with Satterthwaite approximation. Effect sizes are reported as partial eta squared (η*p*^2^).

**Table 7:** Difference-in-differences estimates for uncorrected visual acuity based on logMAR transformation.

Age group	Outcomes	β (DiD)	SE (95% CI)	*T*	*P*
U15	VA-L	−0.066	0.015(−0.096–−0.036)	−4.390	<0.001
	VA-R	−0.055	0.015(−0.084–−0.025)	−3.627	<0.001
U16	VA-L	−0.087	0.015(−0.116–−0.057)	−5.767	<0.001
	VA-R	−0.070	0.015(−0.100–−0.041)	−4.661	<0.001

β (DiD), difference-in-differences estimate; SE, standard error; CI, confidence interval. Negative logMAR values indicate greater improvements in visual acuity in the experimental group relative to the control group. Estimates were derived from linear mixed-effects models adjusted for sex and baseline visual acuity.

## 5 Discussion

This study enrolled 200 Grade 10–11 students (approximately 15–16 years) from Huzhou No. 2 High School in Huzhou, China, using a within-school class-cluster controlled design. Using difference-in-differences and linear mixed-effects models, we evaluated the effects of a 16-week recess-based OMRTP on uncorrected VA and noncycloplegic SE. Overall, the OMRTP was associated with greater improvements in VA compared with routine vision health education alone, supported by significant group × time interactions in the mixed-effects models. For SE, the experimental group showed more favorable short-term functional shifts in noncycloplegic SE relative to the control group, with significant group × time interactions and additional effects of time and eye laterality. Taken together, these findings suggest that a standardized recess-deliverable program may provide a feasible, low-risk school-based visual health support strategy under conditions of high near-work demand.

It should be noted that noncycloplegic refraction was used in the present study. Therefore, short-term changes in SE may reflect not only alterations in refractive status but also fluctuations related to accommodative tension and pseudomyopia (26–28). In this context, a shift in SE toward emmetropia (0 D) is more plausibly interpreted as a short-term functional improvement in accommodative state and visual load, rather than a structural change in refractive error.

From a mechanistic perspective, the effects of the OMRTP can be understood within a hierarchical framework comprising primary, secondary, and supportive pathways. First, the primary pathway likely involves modulation of the accommodative system. During high school years, prolonged and intensive near work imposes substantial accommodative demand, often leading to accommodative lag, instability, and visual fatigue, which may manifest as transient myopic shifts (29). The present far–near alternating visual fixation training may provide periodic relaxation windows for the ciliary muscle, thereby alleviating sustained accommodative load and preventing prolonged accommodative strain. This process may, in turn, contribute to improvements in accommodative function (30). Previous evidence indicates that the ciliary muscle is highly responsive to accommodative demand and may undergo structural adaptation following accommodative or vergence training (31). In addition, reduced accommodative function has been closely associated with visual fatigue and discomfort (30), underscoring the critical role of the accommodative system in visual task performance. Second, a secondary pathway may involve optimization of the oculomotor control system. Structured oculomotor training may enhance fixation accuracy and visuomotor coordination, thereby reducing the need for frequent gaze re-positioning and decreasing inefficient visual load during task execution (32). Prior studies have shown that fixational eye movements enhance the precision of retinal visual encoding (33), and that oculomotor training can improve saccadic accuracy and functional visual performance (32). Together with neuroplastic changes and functional reorganization induced by visual perceptual learning (22), these adaptations may further facilitate more efficient visual task performance. Finally, environmental factors may act as supportive mechanisms, providing a contextual framework that enhances and sustains the effects of the primary and secondary pathways (34, 35). These pathways operate in a coordinated and hierarchical manner, with accommodative regulation as the core driver, supported by oculomotor coordination and modulated by environmental influences.

Under the combined influence of these multi-level mechanisms, the intervention group may exhibit a more stable accommodative state with reduced short-term fluctuations. This may be reflected in noncycloplegic refraction as a shift of SE toward emmetropia. In contrast, the more negative shift in SE observed in the control group is more consistent with the natural progression trend under conditions of sustained academic demand. The directional change in SE, together with the concurrent improvement in VA, supports the notion that the present program primarily enhances visual performance through optimization of functional visual status rather than structural refractive changes. In addition, the presence of a main effect of eye, as well as its interactions with age and group in the SE model, suggests potential interocular differences in refractive or accommodative responses. Such differences may be related to baseline interocular asymmetry, habitual visual behavior, or the sensitivity of noncycloplegic measurements to accommodative fluctuations. However, as variables such as ocular dominance and visual behavior patterns were not explicitly assessed in the present study, these findings should be interpreted as exploratory and warrant further investigation.

From an application perspective, the present program demonstrated good feasibility in school settings, as it can be implemented during recess using standardized procedures with minimal risk. Although conventional near–far visual training primarily focuses on accommodative switching between distances, and other interventions such as oculomotor exercises or increased outdoor exposure have been associated with improvements in visual function, these approaches are typically applied in isolation. In contrast, the OMRTP integrates multiple mechanisms, including accommodative regulation, oculomotor control, and environmental light exposure, within a unified framework. By combining these mechanisms into both active (vision training) and environmental components, the program may provide a more comprehensive visual stimulus than single-modality or conventional near–far training approaches. Consistent with the observed improvements in both VA and noncycloplegic SE, the program may serve as a practical adjunct to routine vision screening and health education. As such, it offers an actionable and scalable strategy to support adolescent visual health under conditions of sustained near-work demand.

### 5.1 Limitations and future directions

Several limitations of the present study should be acknowledged. First, the intervention was implemented using a within-school class-cluster design. Although difference-in-differences was applied to mitigate baseline differences, residual class-level confounding related to classroom environment, teacher factors, or unmeasured behaviors cannot be fully excluded. In addition, a formal *a priori* statistical power analysis was not conducted. The sample size was determined by feasibility within the school setting and included all eligible participants; therefore, the absence of power calculation may limit the precision of effect size estimation. Second, refractive assessments were conducted without cycloplegia. Consequently, changes in spherical equivalent refraction may partly reflect accommodative state and short-term visual fatigue, and should be interpreted as functional rather than structural changes. Furthermore, this study did not include direct structural or mechanistic indicators, such as axial length or accommodative function parameters, and the follow-up duration was relatively short, precluding evaluation of long-term intervention effects.

Future studies should include larger samples across multiple schools and adopt cluster-randomized designs. Incorporating cycloplegic refraction, axial length measurements, and detailed accommodative assessments, together with longer follow-up periods, would enable more robust evaluation of intervention durability and underlying mechanisms.

## 6 Conclusion

The findings of this study suggest that a 16-week school recess-based OMRTP was associated with improvements in uncorrected visual acuity and short-term functional changes in noncycloplegic spherical equivalent refraction among adolescents, with broadly consistent effects across age groups and between eyes.

## Data Availability

The raw data supporting the conclusions of this article will be made available by the authors, without undue reservation.
